# A Clinical Experience

**Published:** 1894-02

**Authors:** Adolph Lippe


### A CLINICAL EXPERIENCE.

Adolph Lippe, M. D.

### Apis-mel. in Typhus.

Mr. J. W., æt. seventeen, being quite indisposed while in the country, became alarmingly ill and hastened to his home, traveling, in this condition, about seventy miles by rail and about ten miles in a carriage. I first saw him December 27th, 1867. I

found him very much prostrated, complaining of violent throbbing headache, with sleeplessness; violent pain in the small of the back; fever high and face very red; aversion to light and noise. The headache and pain in the back were much relieved by a dose of *Belladonna.*

The symptoms most prominent and gradually developing themselves were entire sleeplessness, disinclination to talk, aversion to food, much thirst for cold water. On the fifth day diarrhœa set in. (*Phosphor-acid* had but a short and slight effect on this condition.) The aversion to talk increased; the stools became more frequent; pulse 96; the tongue remained moist and clean till January 6th, when it became dry and red on the tip; the nights were very restless (Rhus-tox. gave no relief). From January 6th the stools passed involuntary. The abdomen was not painful to the touch or pressure. The exacerbation of the fever now commenced at 11 P. M., was at its height about 1 A. M., and diminished at 4 P. M. The pulse became small, hard, and frequent. January 6th he received Arsenicum-alb., and was better on the 7th but worse on the 8th, and a repetition did not produce any effect. The debility increased, the pulse became much smaller, and the knees cold; thirst and the dryness of the tongue increased; complained every day of feeling very nervous. On the 9th he received one dose of Sulphur, but improved for a short time only. On the 11th of January I found him growing worse. The nervousness had now reached a very high degree. The *subsultus tendinum* formerly observed in him but for a short time had now changed into a quivering trembling of the whole body; the extremities could not be kept quiet for a moment. He complained of no pain, but whined continually in the most pitiful manner. The countenance showed great suffering; the features were pinched; aggravation from 11 P. M. till 4 P. M., 1 A. M. being still the height of the aggravation. Frequent involuntary, painless stools; they have been dark brown, papescent, and very offensive, *and are now mixed with mucus;* diarrhœa worse in the morning hours; no *tympanitis;* no pain in the abdomen on pressure; considerable thirst for water; skin dry; lips become blackish, tongue more dry; he lies only on his back. Pulse is now in the neighborhood of 180 to 200. The two principal remedies presenting themselves in the case were *Arsenic* and *Apis*. *Ignatia* had the mental symptom, but did not seem to correspond with the other conditions of the case. Arsenic corresponded well with the time of aggravation, but the characteristic restlessness of Arsenic was not present, but rather complete indifference; the diarrhœa was painless and worse in the morning hours, while the Arsenic diarrhœa is a *painful one*, and is *worse during the night* or *after eating* and drinking. Under Apis we find (Hering's *Amerikanische Arzneipruefungen*) Symptom 1.—*Indifference.* 970. *The whole nervous system seems highly agitated.* 971. *Great irritability of the nerves.* 973 and 1066. *Nervous restlessness the latter part of the night.* 980. *Trembling.* 983. *Trembling of the hands and feet.* 612. *Painless diarrhœa, especially in the morning.* The "quivering-trembling" was the last symptom, not dependent on any former remedy, but clearly indicating the progressiveness of the disease. The totality of the latest and most prominent symptoms was more characteristic of Apis than of any known remedy. On the evening of the 11th of January, the fourteenth day of the disease, the patient received *Apis*$^{20m}$, six pellets dissolved in two ounces of water, a teaspoonful every two hours. The effect was astonishingly happy. The quivering-trembling had ceased during the night, the whining had almost entirely subsided, the diarrhœa was lessened, the pulse in the morning marked 120, full and soft, the tongue less dry. The remedy was continued for three days at longer intervals. The urine was no longer discharged involuntarily, and showed for the first time, when allowed to stand, a cloud in the middle, and later a heavy sediment; perspiration and sleep followed until the patient asked for food on the 18th of January. He had slept for five days almost without interruption. The diarrhœa had ceased entirely, the tongue became moist, and he began to converse again. On the 26th of January he received one dose of Lycopod.$^{10m}$ for a few remaining symptoms, and was fully restored to health without further medication. He returned to college in March, and when I saw him in the last week of April he had gained more flesh, had a better color, was in fuller strength, and better able to pursue his studies than before his illness.—*The Hahnemanian Monthly*, August, 1868.

